# Three-dimensional maxillary virtual patient creation by convolutional neural network-based segmentation on cone-beam computed tomography images

**DOI:** 10.1007/s00784-022-04708-2

**Published:** 2022-09-17

**Authors:** Fernanda Nogueira-Reis, Nermin Morgan, Stefanos Nomidis, Adriaan Van Gerven, Nicolly Oliveira-Santos, Reinhilde Jacobs, Cinthia Pereira Machado Tabchoury

**Affiliations:** 1 Department of Oral Diagnosis, Division of Oral Radiology, Piracicaba Dental School, University of Campinas (UNICAMP), Av. Limeira 901, Piracicaba, São Paulo 13414‑903 Brazil; 2 OMFS IMPATH Research Group, Department of Imaging & Pathology, Faculty of Medicine, KU Leuven & Oral and Maxillofacial Surgery, University Hospitals Leuven, Kapucijnenvoer 33, 3000 Leuven, Belgium; 3 Department of Oral Medicine, Faculty of Dentistry, Mansoura University, Mansoura , 35516 Dakahlia Egypt; 4 Relu BV, Kapeldreef 60, 3000 Louvain, Belgium; 5 Department of Dental Medicine, Karolinska Institutet, Box 4064, 141 04 Huddinge, Stockholm Sweden; 6 Department of Biosciences, Division of Biochemistry, Piracicaba Dental School, University of Campinas (UNICAMP), Av. Limeira 901, Piracicaba, São Paulo 13414‑903 Brazil

**Keywords:** Computer simulation, Three-dimensional image, Artificial intelligence, Computational neural networks, Cone-beam computed tomography, Jaw bone, Tooth

## Abstract

**Objective:**

To qualitatively and quantitatively assess integrated segmentation of three convolutional neural network (CNN) models for the creation of a maxillary virtual patient (MVP) from cone-beam computed tomography (CBCT) images.

**Materials and methods:**

A dataset of 40 CBCT scans acquired with different scanning parameters was selected. Three previously validated individual CNN models were integrated to achieve a combined segmentation of maxillary complex, maxillary sinuses, and upper dentition. Two experts performed a qualitative assessment, scoring-integrated segmentations from 0 to 10 based on the number of required refinements. Furthermore, experts executed refinements, allowing performance comparison between integrated automated segmentation (AS) and refined segmentation (RS) models. Inter-observer consistency of the refinements and the time needed to create a full-resolution automatic segmentation were calculated.

**Results:**

From the dataset, 85% scored 7–10, and 15% were within 3–6. The average time required for automated segmentation was 1.7 min. Performance metrics indicated an excellent overlap between automatic and refined segmentation with a dice similarity coefficient (DSC) of 99.3%. High inter-observer consistency of refinements was observed, with a 95% Hausdorff distance (HD) of 0.045 mm.

**Conclusion:**

The integrated CNN models proved to be fast, accurate, and consistent along with a strong interobserver consistency in creating the MVP.

**Clinical relevance:**

The automated segmentation of these structures simultaneously could act as a valuable tool in clinical orthodontics, implant rehabilitation, and any oral or maxillofacial surgical procedures, where visualization of MVP and its relationship with surrounding structures is a necessity for reaching an accurate diagnosis and patient-specific treatment planning.

**Supplementary Information:**

The online version contains supplementary material available at 10.1007/s00784-022-04708-2.

## Introduction

One of the recent trends for diagnostics and pre-surgical planning in orthodontics, orthognathic surgery, and oral implant placement has been the introduction of simplified digital workflows [1]. The solid basis of such workflows can often be accomplished by three-dimensional (3D) imaging, mainly cone-beam computed tomography (CBCT), which offers volumetric anatomical data of orofacial structures.

Segmentation of the imaging data acquired from CBCT is essential for generating 3D models of patient-specific anatomical structures, which is a prerequisite for virtual treatment planning and 3D manufacturing [1]. However, current segmentation techniques, either manual or semi-automatic, are time-consuming, suffer from human variability, and are hampered by metal and motion artifacts [2]. Besides, segmentation of CBCT images requires more time than traditional multi-slice computed tomography (MSCT), as MSCT images have a superior contrast resolution and lower noise which facilitate achieving a time-efficient segmentation [2–4]. Nevertheless, CBCT acts as the modality of choice in oral healthcare, considering its low cost, relatively lower dose, and increased accessibility [2, 5].

Considering these limitations of CBCT imaging in relation to segmentation, there is a need for automation of the current digital workflows by the application of artificial intelligence (AI)-based techniques. Recently, a convolutional neural network (CNN), a class of artificial neural networks, has dominated the field of medical image analysis, as it is specialized for processing data with defined, grid-like topology, such as two-dimensional (2D) and 3D images [6, 7]. CNNs have the ability to outperform standard image processing algorithms with high computational speed and correlate with other data such as clinical information or response to therapy. This provides an improvement in the quality of image processing and helps clinicians to extract and analyze relevant information in a concise format [7].

So far, the authors of several studies have focussed on the segmentation of individual craniomaxillofacial anatomical structures using CNN models [8–11]. However, no evidence exists about the integration of these multiple anatomical structures as a single unit. A combination of AI models specialized in segmenting different structures with variable densities simultaneously could pave the way towards the creation of a virtual patient with high performance in a time-efficient approach. This virtual patient could be applied for digital virtual planning of several treatment procedures, not only in general dentistry but also in maxillofacial surgery; Ear, Nose, and Throat (ENT); neurosurgery; and ophthalmology. Therefore, we aimed to assess the qualitative and quantitative performance of integrated CNN models of three previously validated individual networks for the creation of a segmented maxillary virtual patient (MVP) consisting of maxillary skeletal complex, maxillary sinuses, and teeth from CBCT images [8, 12, 13]. We hypothesized that the three integrated CNN models would reveal a similar performance as the individuals’ ones, along with a strong interobserver agreement in terms of time-efficiency and consistency for creating a segmented MVP.”

## Materials and methods

This study was approved by the Research Ethics Committee of the University Hospitals Leuven (reference number: S65708) and was conducted in compliance with the World Medical Association Declaration of Helsinki on medical research. Patient-specific information was anonymized.

### Dataset

The sample size was calculated based on previous comparable studies using a priori power analysis in G* power 3.1, with a power of 80% and a significance level of 5%[9, 11]. In this way, a total dataset of 40 scans of two devices (20 *Accuitomo 3D*; 20 *Newtom VGi evo*) was selected, consisting of 560 teeth, 80 sinuses, and 40 maxillofacial complexes acquired with different scanning parameters (Table 1). Inclusion criteria were scans with permanent dentition, including teeth with coronal and/or root fillings. Exclusion criteria were patients with a history of maxillofacial trauma, skeletal or dental malformation, post-orthognathic surgery patients with mini-plates and screws, presence of dental implants, and missing teeth in proximity to the sinus floor.

**Table 1:** CBCT scanning parameters of the sample

	kVp	mA	Voxel size (mm)	Field of view (cm)
Newtom VGi evo (Cefla, Imola, Italy)	110	3–8	0.2; 0.25; 0.3	24X19; 16X16; 12X8; 10X10
3D Accuitomo 170 (J. Morita, Kyoto, Japan)	90	5	0.25	17X12; 14X10; 10X10; 8X8

*kVp* kilovoltage peak, *mA* milliampere

All CBCT images were saved in Digital Imaging and Communication in Medicine (DICOM) format and uploaded to an online cloud-based platform called Virtual Patient Creator (creator.relu.eu, version December 2021, Relu BV, Leuven, Belgium), which allowed combined automatic segmentation of maxillary complex, maxillary sinuses, and teeth, referred to as MVP.

### Qualitative assessment

**Fig. 1:**
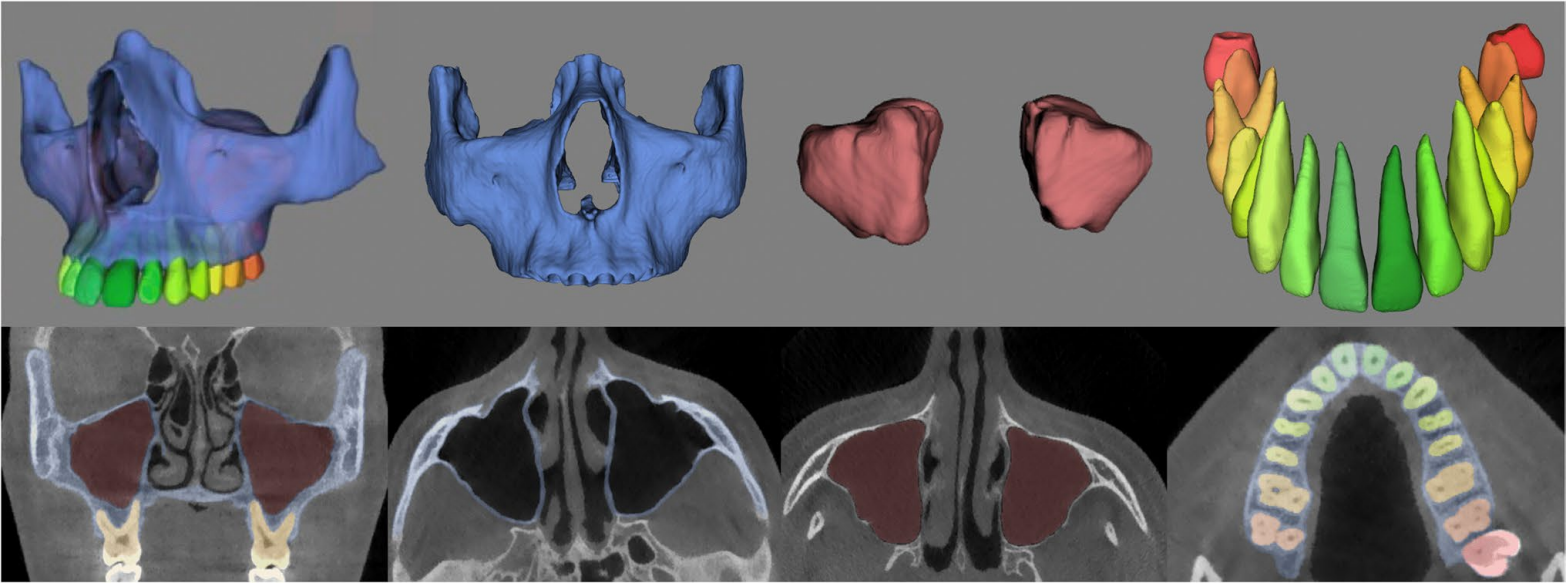
3D views and their respective colored segmentations on CBCT slices on Virtual Patient Creator (creator.relu.eu, Relu BV, Version December 2021): **a** all structures combined showing the maxillary virtual patient, **b** maxillofacial complex, **c** maxillary sinuses, and **d** upper dentition

**Fig. 2:**
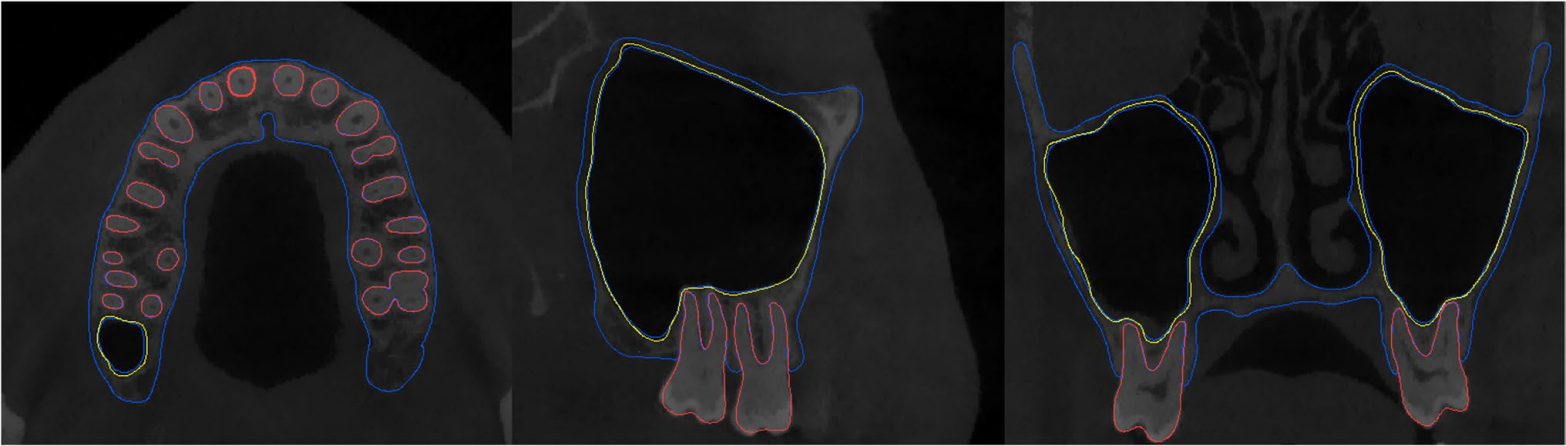
Borderline definition of the automatic segmentation of integrated structures (version 23.0, Materialise N.V., Leuven, Belgium)

Two dentomaxillofacial radiologists (FNR and NM) clinically evaluated the automatic segmentation of the integrated structures by visually observing their corresponding colors on orthogonal planes of the CBCT images (Fig. 1). The three individual CNN models of maxillofacial complex, maxillary sinuses, and teeth have been previously validated, where they were proved to be highly accurate, requiring only minor refinements (slight over or under segmentation in each structure) (Fig. 2). Hence, a score from 0 to 10 was given for each segmentation based on the number of required minor refinements, where 0 represented ten refinements or more, 1 represented 9 refinements, 2 represented 8 refinements, and successively up to 10 that referred to a perfect segmentation without the need for any refinement. Inter-observer agreement was assessed for the scoring between the two observers. Additionally, needed refinements were performed for assessing the performance of the integrated models in comparison to the refined ones, and the consistency between observers.

### Smart correction tools

Following visual assessment, both observers performed the required refinements using the newly developed tools on the virtual patient creator platform: normal and smart brushes, contour, and livewire tools. The normal brush is a simple cylindrical brush, which is used for adding brush strokes to refine small inadequacies between multiple image slices. The smart brush uses voxel intensities to group them by analyzing the voxel’s intensity below the cursor and selecting all voxels at a certain depth that have intensities within the selected voxel’s tolerance range. Both tools are unidirectional, causing only the slices above or below to be changed. Hence, there was no issue of overwriting slices that have already been corrected.

The contour tool automatically interpolates the inter-slice region between upper and lower selected contours. The livewire tool is an intelligent version of the contour tool, whose main principle of inter-slice interpolation remains the same. However, it connects the added points in a path that automatically follows the grey values of the image. Consequently, allowing the user to outline contours more quickly with a fewer number of points compared to a contour tool. Tutorials on how to use these tools are available as supplementary material (online resources 1–4).

### Quantitative assessment

#### Timing

The time required to have a full-resolution automatic segmentation (AS) was measured directly by an automated algorithm. As for the refined segmentation (RS), it was calculated by summing up the time required for automatic segmentation and refinements. Finally, the average time for each segmentation technique was calculated.

#### Automatic versus refined segmentations

The automatic segmentation was compared to the manual refined segmentation, and the metrics used to assess its similarity included dice similarity coefficient (DSC), 95% Hausdorff distance (HD), and root mean square (RMS) (Table 2). The performance of the AI models for MVP segmentation was calculated using the following expression, where *x* is the comparison metric of interest (e.g., DSC) between automatic and refined segmentation.

**Table 2:** Overview table of validation metrics used in the quantitative assessment

Metrics	Definition	Formula
DSC	This ratio represents how similar the segmented region is to the ground truth	*2*|*A* ∩ *B*|*2* × *TP* *DSC* (*A*, *B*) = |*A*| +|*B*| = *2* × *TP* + *FP* + *FN*
95% HD	Indicator of the maximum difference between the limits of the automatic segmentation and the ground truth	95% HD = *P*_95_(min||*B* − *A*||_2_ U min||*A* − *B*||) *a*g*Ab*g*B*2
RMS	Indicator of the imperfection of the fit between the STLs of the surface of interest and ground truth in mm	*1* *RMS* = √ = (*d*^*2*^ + *d*^*2*^ + ... *d*^*2*^) _*n*_*12n*

*DSC* Dice Similarity Coefficient, *95%HD* 95% Hausdorff distance, *RMS* root mean square, *A* volumetric data of observer 1, *B* volumetric data of observer 2, *TP* true positives, *TN* true negatives, *FP* false positives, *FN* false negatives, *P*_*95 *_percentile 95

$$\chi\;_{combined}=\frac{\langle x\;_{maxillofacial\;complex}\rangle+\langle x\;_{maxillary\;sinuses}\rangle+\langle x\;_{upper\;dentition}\rangle}3$$

The dentition metric was defined as the average overall individual tooth types:

$${\chi }_{upper dentition}= \frac{\langle {x}_{tooth 11}\rangle +\langle {x}_{tooth 12}\rangle +\dots }{16}$$

#### Consistency of refined segmentations

The three CNN models have already proven to be 100% consistent at an individual level, hence AI consistency was not further investigated. The interobserver consistency of refined segmentations was assessed by overlapping the DICOM and resultant STL files of the segmentations performed by each observer. Thereafter, corresponding evaluation metrics were calculated.

### Statistical analysis

Data were analyzed with IBM SPSS version 28.0.1.0 software (Armonk, NY). The weighted Kappa test (95%CI) was performed for the inter-observer agreement of the qualitative assessment. For quantitative data, the mean value and standard deviation of each evaluation metric were calculated.

## Results

**Fig. 3:**
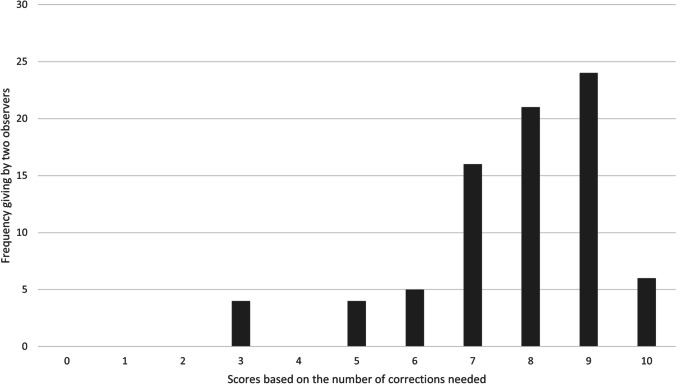
Score frequency based on the number of corrections needed given by two observers (*n* = 80)

**Fig. 4:**
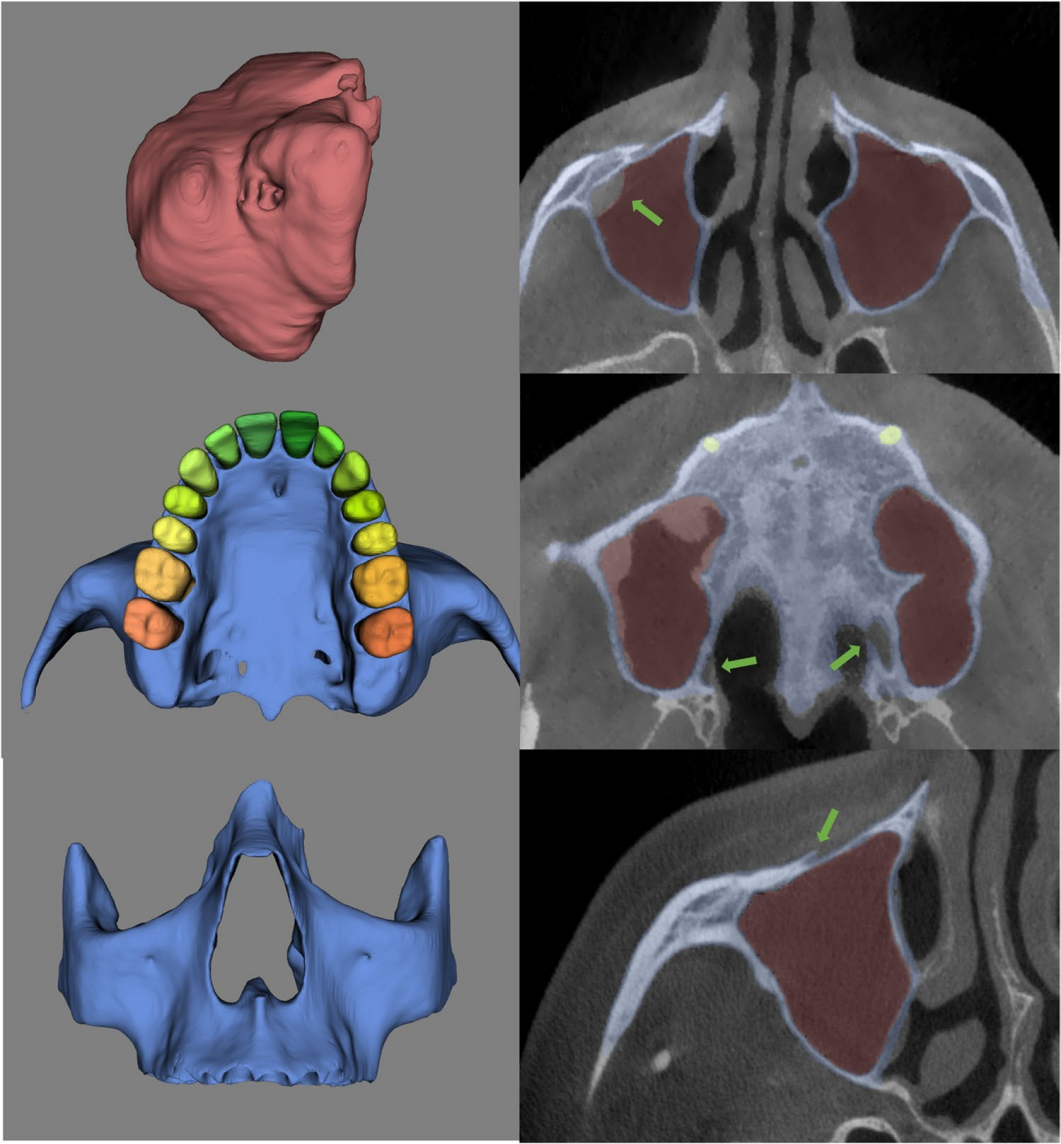
3D models and axial sections of CBCT scans illustrating the necessary refinements most often detected in qualitative analysis. **a** Mucosal thickening in the upper cortical of the right maxillary sinus. **b** MVP in a view showing bone discontinuity around palatine foramina. **c** Closed right infraorbital foramen in a lateral view of the MVP

Based on the visual assessment, there was no overlap between the three structures (Fig. 2). From the entire dataset, 85% showed a score of 7 or more by both observers, and 15% were within the range of 3–6. Furthermore, there were no cases with scores of 0–2 (Fig. 3). In total, 40 scans required minor corrections, mainly due to mucosal thickening in the sinus, closed foramina and canals, small bone discontinuities in the palate and maxilla, and bone over-segmentation of zygomaticotemporal sutures (Table 3). Figure 4 illustrates some examples of the regions requiring refinements. The weighted Kappa test showed a strong inter-observer agreement (*K* = 0.832, 95% CI [0.704;0.960]) based on Landis and Koch’s classification [14].

**Table 3:** Types of corrections required according to the structure with their description

Structure refined	Correction type	Description
Upper dentition	Under segmentation	Small missing parts in the tooth contour
	Over segmentation	Not found
Maxillary sinuses	Under segmentation	Mucosal thickening, and air voids
	Over segmentation	Overextension in ethmoidal air sinus
Maxillofacial complex	Under segmentation	Bone discontinuities in the medial wall and back of the maxilla, and in the palate
	Over segmentation	Closed infraorbital and palatine foramina, nasopalatine canal, and overextension of zygomaticotemporal suture

The average timing for the automated segmentation of 40 cases was 1.7 min, ranging from 1.1 to 2.4 min. The average time required for refinements by the first and seconds was 3.4 min (1.2 to 15 min) and 2.5 min (1.0 to 11 min), respectively.

The performance metrics (Table 4) indicated an excellent overlap between automatic and refined segmentation with a DSC of 99.3% for both observers, implying that minimal refinements were required. The RMS value was 0.289 mm and 0.286 mm, and the 95% HD was 0.210 mm and 0.228 mm for each observer, respectively.

**Table 4:**
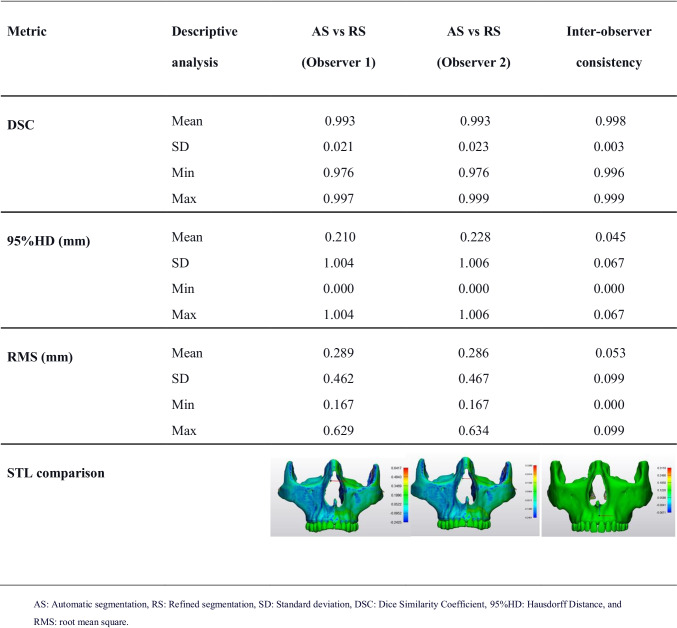
Evaluation metrics for comparison between automatic and refined segmentations

Interobserver consistency of refinements (Table 4) showed a high DSC of 99.8%. A close to zero 95% HD of 0.045 was detected with a low RMS value of 0.053. Additionally, the STL overlap comparison map also observed a similar pattern, hence suggesting a substantial agreement between both observers.

## Discussion

An accurate 3D segmentation of orofacial structures is the first essential step in most digital dental workflows. It is crucial for precise delineation and outlining of normal anatomy, variations, differentiation from accompanied pathological lesions, and volumetric estimation of anatomical structures. If segmentation of multiple anatomical structures is performed simultaneously, it provides a clinician with a complete picture and focused approach towards studying the relation with the surrounding structures. Therefore, the present study investigated the performance of integrated CNN models for creating the MVP consisting of combined automatic segmentation of the maxillary complex, sinus, and teeth as a single unit.

For qualitative assessment, since only minor corrections were needed, the quality of integration was assessed based on the number of refinements and the required time. The results showed a strong agreement between both observers. A score equal to 7 or more (85% of the dataset) was considered a high-quality segmentation, while a score ranging from 3 to 6 (15% of the dataset) an above-average quality. Segmentations in Table 3 illustrate the types of required refinements per segmented structure. According to previous validation studies’ classification [12, 13], minor refinements have no or slight clinical relevance, and the present qualitative analysis assumes that this clinical impact depends on the number of minor refinements needed. In daily practice, the clinical relevance of such refinements might differ depending on the task at hand, such as visualization, diagnosis, treatment planning, and patient education. Moreover, each type of refinement might be more relevant in a specific clinical specialty compared to another one. For instance, mucosal sinus thickness is more relevant for treatment planning in oral and maxillofacial surgical procedures involving maxillary sinus floor elevation [15] compared to a routine dental examination or patient education.

The quantitative assessment revealed that the sum of mean time required for automatic MVP segmentation (1.7 min) was slightly higher compared to the sum of the previously documented timing for each structure segmentation which totaled 1.3 min (maxillofacial complex: 39.1, maxillary sinus: 24.4, all teeth: 13.7 s) [8, 12, 13]. This minimal difference could be attributed to some technical variabilities, such as nonuser active processes, which impact the segmentation time even if the same AI tool is run several times, making it a challenge to keep the time constant [16]. Another reason could be the large field of view (FOV) of the included sample, which could have increased the processing time. The previous studies used fewer testing samples with large FOVs because they covered only one region of interest.

We did not investigate the clinical accuracy of automated segmentation, which has previously been reported to have a high DSC score (maxillary complex: 92.6%, maxillary sinus: 98.4%, teeth: 90%), when compared to the reference ground truth generated by skilled human operators using a manual or semi-automatic approach. Rather, the relevant performance of the combined structural segmentation was compared to the manually refined one. The findings showed no change in performance following post-integration. A DSC score of 99.3% was observed compared to refined segmentation for both observers, hence implying high segmentation quality even for the scans requiring many refinements. Additionally, the interobserver consistency showed almost perfect overlap with a DSC of 99.8%, indicating that the integrated model could provide an automated ground that increases consistency between observers overcoming high observer variability in other segmentation techniques.

The presented CNN model overcame the issue of manual threshold selection required with semi-automatic approaches. Moreover, the main benefit of the model is the simultaneous segmentation of anatomical structures with different densities using a single platform, as shown in the coronal slice of Fig. 1a. This type of combined segmentation is not possible with the available semi-automatic segmentation software programs, where each structure has a different threshold requiring manual adjustment separately by the operator [17]. Clinically, this integrated segmentation could be a valuable tool in clinical orthodontics and maxillofacial surgical procedures, such as implant planning, bone grafting, and orthognathic and reconstructive surgery [18–21], where visualization of MVP and its relationship with surrounding structures is a necessity for reaching an accurate diagnosis and patient-specific treatment planning.

An additional advantage of the proposed approach was that no third-party software was required to refine the automated segmentations, which was not the case in the previous individual CNN model-based validation studies. As newly developed tools have been employed on the platform, which also let the clinicians directly refine the segmentations. However, lack of data heterogeneity remains a limitation, and there is a need to incorporate data from other CBCT devices with varying scanning parameters to justify the generalizability of the tool. In the near future, we plan to integrate other validated individual anatomical regions, such as the mandible, inferior alveolar canal, and pharyngeal airway [9–11]. It is also expected to expand the tool’s ability by integrating data from intra-oral scanners and facial scanners for the creation of a complete virtual patient, which could enhance the delivery of personalized dental care [22]. Furthermore, additional CBCT scans from various institutions, CBCT scanner brands, and the variability of patient anatomy and pathology should be integrated in the near future to increase the generalizability further. The application of AI tools and personalized data in clinical and research fields could support positive clinical protocols changes, help create predictive population models [23], and act as a visual educational tool for both clinicians and patients.

## Conclusion

The three integrated CNN models proved to be fast and accurate for simultaneous segmentation of maxillary anatomical structures with different densities. Both the qualitative and the quantitative assessments revealed a strong interobserver consistency. The integrated MVP could act as a feasible tool for visualization, diagnostics, and treatment planning in daily clinical practice.

## Supplementary Information

Below is the link to the electronic supplementary material.

Supplementary file1 (MP4 96032 KB)
